# Functional Rescue of Trafficking-Impaired ABCB4 Mutants by Chemical Chaperones

**DOI:** 10.1371/journal.pone.0150098

**Published:** 2016-02-22

**Authors:** Raquel Gordo-Gilart, Sara Andueza, Loreto Hierro, Paloma Jara, Luis Alvarez

**Affiliations:** 1 La Paz University Hospital Health Research Institute-IdiPAZ, Madrid, Spain; 2 Pediatric Liver Service, La Paz Children’s University Hospital, Madrid, Spain; Simon Fraser University, CANADA

## Abstract

Multidrug resistance protein 3 (MDR3, ABCB4) is a hepatocellular membrane protein that mediates biliary secretion of phosphatidylcholine. Null mutations in *ABCB4* gene give rise to severe early-onset cholestatic liver disease. We have previously shown that the disease-associated mutations p.G68R, p.G228R, p.D459H, and p.A934T resulted in retention of ABCB4 in the endoplasmic reticulum, thus failing to target the plasma membrane. In the present study, we tested the ability of two compounds with chaperone-like activity, 4-phenylbutyrate and curcumin, to rescue these ABCB4 mutants by assessing their effects on subcellular localization, protein maturation, and phospholipid efflux capability. Incubation of transfected cells at a reduced temperature (30°C) or exposure to pharmacological doses of either 4-PBA or curcumin restored cell surface expression of mutants G228R and A934T. The delivery of these mutants to the plasma membrane was accompanied by a switch in the ratio of mature to inmature protein forms, leading to a predominant expression of the mature protein. This effect was due to an improvement in the maturation rate and not to the stabilization of the mature forms. Both mutants were also functionally rescued, displaying bile salt-dependent phospholipid efflux activity after addition of 4-PBA or curcumin. Drug-induced rescue was mutant specific, given neither 4-PBA nor curcumin had an effect on the ABCB4 mutants G68R and A934T. Collectively, these data indicate that the functionality of selected trafficking-defective ABCB4 mutants can be recovered by chemical chaperones through restoration of membrane localization, suggesting a potential treatment for patients carrying such mutations.

## Introduction

Multidrug resistance protein 3 (MDR3, ABCB4) deficiency is among those liver disorders associated with impairment of the canalicular transport of bile constituents [1]. ABCB4 is an ATP-binding cassette (ABC) transmembrane protein that translocates phosphatidylcholine (PC) from the inner to the outer leaflet of the canalicular membrane of hepatocytes [2–4]. By doing so, it makes possible the biliary secretion of PC. Genetic alterations of *ABCB4* gene result in ABCB4 deficiency and hence in the formation of PC-deprived bile, which produces hepatobiliary injury [5]. The severity of the liver disease in ABCB4 deficiency ranges from biochemical abnormalities with annoying symptoms to life-threatening disease with the potential for progression to death due to hepatic failure [6]. The varying clinical phenotypes are in part related to the extent to which ABCB4 function is impaired by particular mutations. Patients carrying *ABCB4* defects that result in a complete loss of ABCB4 function display progressive liver damage that typically requires liver transplantation during childhood. Patients with milder missense mutations or with heterozygous defects exhibit less severe phenotypes and might respond to ursodeoxycholic acid (UDCA) therapy [5,6]. To date, 35 *ABCB4* disease-causing missense variants have been characterized *in vitro* [7–17]. They preclude proper functioning of ABCB4 in various ways, affecting trafficking of the protein to the plasma membrane, protein expression, or PC-translocating activity. Nine out of these 35 variants cause intracellular retention of ABCB4 in the endoplasmic reticulum (ER), leading to mislocalization to the plasma membrane [7,8,11,13,16,17]. Such as mutations clearly result in a loss-of-function protein, and biallelic carriers are expected to develop progressive, end-stage liver disease [8,13].

In recent years, strategies to ameliorate the consequences of mutations in genes encoding hepatocanalicular transporters have shown some promise [18,19]. Drugs with chaperone-like activity are being tested to deal with ER-trapped mutant proteins, because these compounds might facilitate the folding and transport to the plasma membrane of misfolded, intracellularly retained proteins [20]. Molecules such as 4-phenylbutyrate (4-PBA) or curcumin, known as “chemical chaperones”, have been shown to rescue *in vitro* mislocalized mutant ABC transporters and canalicular membrane proteins, including ABCA1, ABCA3, ABCB11 (BSEP), ABCC6, ABCC7 (CFTR), ABCD2, ATP7B and ATP8B1 [21–30]. Beneficial effects of 4-PBA treatment have also been reported for patients bearing ER-retained ATP8B1 and BSEP protein mutants [31–35].

We recently reported that the *ABCB4* mutations p.G68R, p.G228R, p.D459H, and p.A934T, which we found in children with progressive familial intrahepatic cholestasis type 3 (PFIC3), resulted in ABCB4 protein being trapped in the ER [13,16]. In this study, we investigated whether all these mutant proteins can be targeted by chaperone drugs and restore plasma membrane localization and activity. Our data indicated that some of these mutants could be functionally rescued by such compounds, providing a pharmacological option for the treatment of specific patients.

## Materials and Methods

### Plasmids and Reagents

The generation of the ABCB4 mutants G68R, G228R, D459H and A934T has been previously described [13,16]. Madin-Darby canine kidney MDCK-II cells and human embryonic kidney AD-293 cells were obtained from ATCC (LGC Standards Barcelona, Spain) and Agilent Technologies (Santa Clara, CA, USA), respectively. The mouse monoclonal anti-ABCB4 antibody (clone P3II-26) was purchased from Millipore (Billerica, Masachussets, USA). The rabbit anti-calnexin antibody was obtained from StressMarq (Victoria, Canada), and the anti-Na/K-ATPase antibody was obtained from Santa Cruz Biotechnology (Santa Cruz, CA, USA). Anti-mouse AlexaFluor594-conjugated and anti-rabbit AlexaFluor488-conjugated secondary antibodies were from Molecular Probes (Eugene, OR, USA). Sodium 4-PBA, curcumin, cycloheximide, brefeldin A and standard lipids (phosphatidic acid, phosphatidylinositol, phosphatidylcholine, lysophosphatidylcholine, phosphatidylethanolamine, phosphatidylserine, sphingomyelin) were provided by Sigma (St. Louis, MO, USA). [^3^H]-choline (60–90 Ci/mmol) was purchased from Perkin Elmer (Massachusetts, USA). All other reagents were of analytical grade.

### Cell Culture, Transient Transfections, and Treatments

The cells were cultured in Dulbecco's modified Eagle's medium supplemented with 10% fetal bovine serum, 2 mM L-glutamine, 100 U/ml penicillin, and 100 μg/ml streptomycin at 37°C in a humidified atmosphere with 5% CO_2_. Transient transfections were performed as previously described [13]. The experiments were carried out 24 h after transfection. To assess the effect of temperature, MDCK-II cells were maintained at 37°C or shifted to 30°C for a further 24 h before immunofluorescence. For drug treatments, MDCK-II and AD-293 cells were incubated for 24 or 48 h in the presence of various concentrations of sodium 4-PBA (1, 2, 4 mM) or curcumin (1, 2.5, 5 μM). Curcumin and 4-PBA were dissolved in DMSO and H_2_O, respectively. To inhibit protein synthesis, cycloheximide was added to transfected AD-293 cells at a final concentration of 50 μg/ml. For trafficking inhibition, transfectants were treated with 1 μg/ml brefeldin A for 2 h.

### Immunofluorescence Staining and Confocal Microscopy

MDCK-II cells were plated on coverslips and transfected with wild-type or respective mutant ABCB4-expressing plasmids. Twenty-four or 48 h after treatments, the cells were fixed for 10 min with 2% paraformaldehyde and permeabilized for 40 min with 0.075% saponin in 10% fetal bovine serum. Fixed, permeabilized cells were incubated with the primary antibodies (mouse monoclonal anti-ABCB4, dilution 1:100; and rabbit polyclonal anti-calnexin, dilution 1:500) for 90 min at room temperature. The cells were washed and incubated with anti-mouse AlexaFluor594-conjugated and anti-rabbit AlexaFluor488-conjugated secondary antibodies at 1:200 dilution. Images of the stained cells were obtained using a Leica TCS-SP2 laser scanning confocal microscope.

### Western Blotting

AD-293 cells were lysed in ice-cold PBS containing 1% Nonidet P-40, 0.5% sodium deoxycholate, 0.1% SDS and 1% protease inhibitor cocktail (Roche Applied Science, Indianapolis, IN). Equal amounts of total proteins (30 μg) were separated by 6% SDS-PAGE and transferred onto PVDF membranes. For immunochemical detection, the blots were sequentially probed with anti-ABCB4 and anti-Na/K-ATPase antibodies at a 1:20,000 and 1:3,000 dilution, respectively. Horseradish peroxidase-conjugated secondary antibodies (DakoCytomation, Glostrup, Denmark) were used at a 1:20,000 dilution. The signal was developed using the ECL Advanced Western Blotting Detection Kit (GE Healthcare, Buckinghamshire, UK). Quantification of band intensity was performed with ImageJ software (NIH, Bethesda, MD, USA).

### Real-time Quantitative PCR

RNA was isolated from AD-293 cells using the RNeasy Mini kit (Qiagen, Valencia, CA, USA) and subsequently treated with RNase-free DNase I (Ambion Inc., Austin, TX, USA). Complementary DNAs (cDNAs) were synthesized from 0.5 μg of RNA using AMV (Roche Applied Science) and random hexamers. Real-time quantitative PCR was performed as previously described [13].

### [^3^H]-PC Efflux Assay

Phospholipid efflux assays were performed using transfected AD-293 as previously described [13]. Twenty-four hours after transfection, the cells were treated with 1 mM 4-PBA or 1 μM curcumin and incubated for an additional 24 h. Next, cells were starved for 1 h at 37°C in serum-free, choline low Minimal Essential Medium (EMEM) before being labeled for 3 h with 2 μCi/ml [^3^H]-choline in EMEM. The cells were then washed twice and incubated for 3 h in EMEM in the presence or absence of 1mM sodium taurocholate (NaTC). Aliquots of the culture media (50 μl) were removed each 30 min and the radioactivity released was quantified by liquid scintillation counting. After the last removal, the cells were lysed for mRNA isolation and assessment of protein concentration. Radioactivity values were corrected for transfection efficiency using measurements of mRNA levels, and were expressed relative to the cell protein. Data shown are from four independent experiments carried out in duplicate. The statistical analysis was performed using the unpaired Student *t*-test.

### Analysis of labeled lipids

AD-293 cells were transfected with the wild-type *ABCB4* cDNA, labeled with [^3^H]-choline and challenged with NaTC as detailed above. After incubation for 3 h, the medium (10 ml) was collected and centrifuged to remove cell debris. An aliquot of 100 μl was used to estimate the total radioactivity released. The lipids were extracted from the medium with chloroform-methanol (2:1). The organic and aqueous phases were separated and dried under nitrogen and vacuum, respectively. The radioactivity associated with the aqueous fraction was counted following addition of scintillation fluid. Lipids present in the organic phase were analyzed essentially as described [36]. Briefly, they were separated by thin layer chromatography on silica gel 60 plates using chloroform-methanol-28% ammonia (65:35:5 v/v) as the running solvent. Lipid standards were run on each plate. Individual lipid components were visualized by iodine vapor. The spots were scraped off and the radioactivity was quantified in a liquid scintillation counter.

## Results

**Fig 1:**
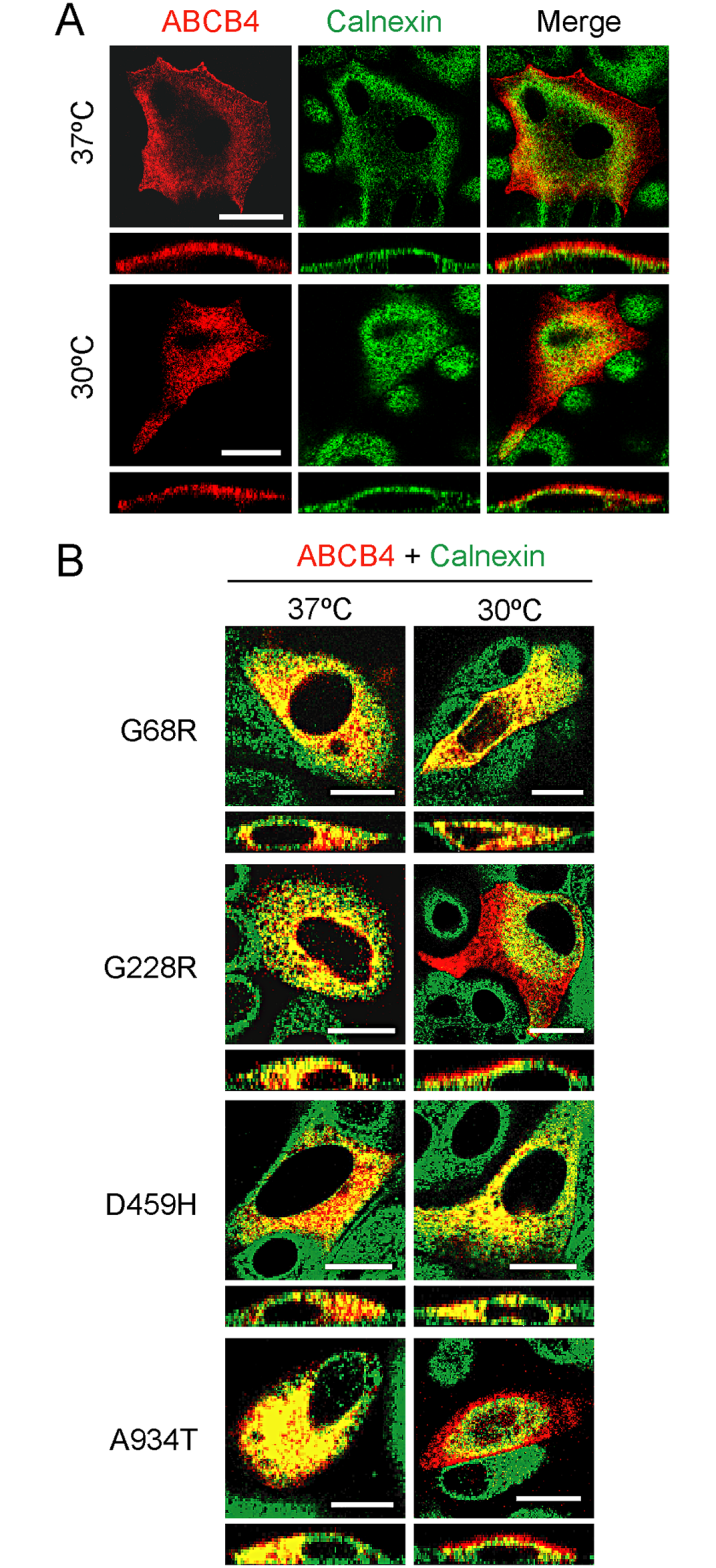
Low temperature restores apical membrane localization of particular ABCB4 mutants. **(A)** Representative double immunofluorescence staining for ABCB4 (red) and the ER marker calnexin (green) in MDCK-II cells expressing wild-type ABCB4 after incubation at 37°C or 30°C. Areas of yellow in the merged images indicate colocalization of ABCB4 and calnexin. Optical horizontal x,y (upper) and vertical x,z sections (lower) are shown. **(B)** Confocal microscopy images of cells expressing ABCB4 mutants. For simplicity, only the merged images are shown. Reducing temperature improved cell surface localization for the mutants G228R and A934T, as revealed by the appearance of red signal around the apical membrane. All images represent three independent transfections. Bars, 20 μm.

In previous studies we showed that the disease-associated ABCB4 mutants G68R, G228R, D459H, and A934T failed to target the apical membrane and localized in the ER when expressed in polarized MDCK-II cells [13,16]. To investigate whether the surface location of these ABCB4 mutants could be restored, we first examined the effects of culturing the cells at low temperature, a treatment that can correct the trafficking of ER-retained misfolded membrane proteins [37]. MDCK-II cells were transfected with wild-type or mutant ABCB4-expressing plasmids and incubated at 37°C or 30°C before being double stained for ABCB4 and the ER marker calnexin. Confocal microscopy showed that low-temperature incubation of cells for 24 h did not affect the localization of the wild-type protein (Fig 1A). The four ABCB4 mutants completely localized in the ER when the cells were cultured at 37°C, as visualized by colocalization with calnexin (Fig 1B). When the temperature was reduced, the localization of the G228R and A934T mutants partially shifted from the ER to the apical surface, whereas the G68R and D459H mutants remained in the ER (Fig 1B). These results suggested that impaired trafficking of at least two of the four ABCB4 mutants could be corrected.

**Fig 2:**
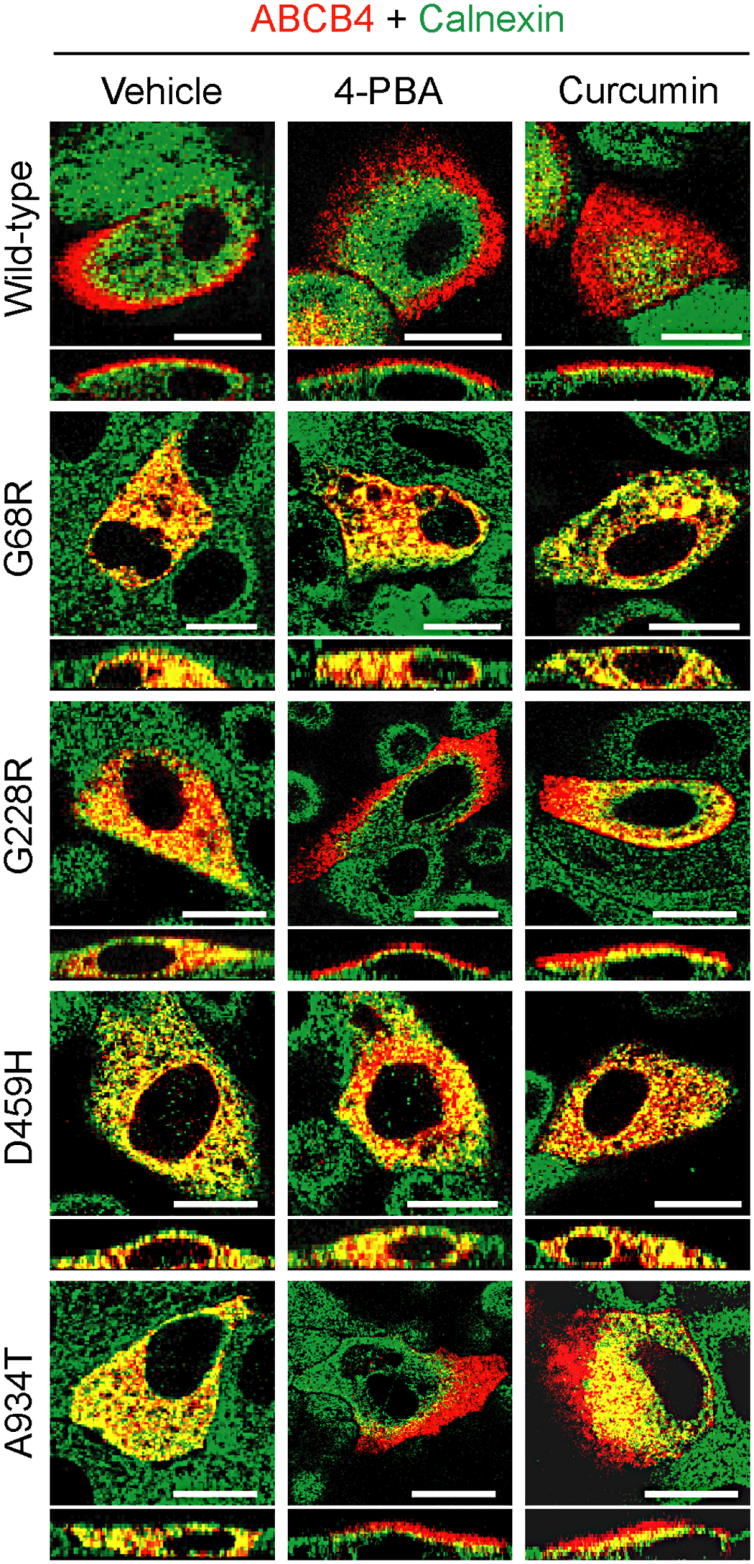
Chemical chaperones rescue cell surface expression of the ABCB4 mutants G228R and A934T. Transfected MDCK-II cells were treated with 0.1% DMSO (vehicle), 1 mM 4-PBA or 1 μM curcumin during 24 h. The cells were double stained for ABCB4 and calnexin and analyzed by confocal microscopy. The merged images of ABCB4 and calnexin are presented. Co-localization (yellow) of calnexin with G68R or D459H mutants was seen after addition of any of the chaperone drugs. Positive apical staining for ABCB4 (red) was found in cells expressing the G228R and A934T mutants following treatment with either 4-PBA or curcumin. The images are representative of four independent experiments, in which at least two different batches of each plasmid were used. Bars, 20 μm.

Transfected MDCK-II cells were then exposed to two common chemical chaperones, 4-PBA and curcumin, at pharmacologically achievable doses. As shown in Fig 2, treatment with either 1 mM 4-PBA or 1 μM curcumin for 24 h rescued the cell surface expression of the ABCB4 mutants G228R and A934T, but failed to restore trafficking of the G68R and D459H mutants to the apical membrane. The mislocalization of these two mutant proteins did not improve by increasing the concentration of drugs (2 mM and 4 mM 4-PBA; 2.5 μM and 5 μM curcumin) or when treatment was prolonged to 48 h (data not shown).

**Fig 3:**
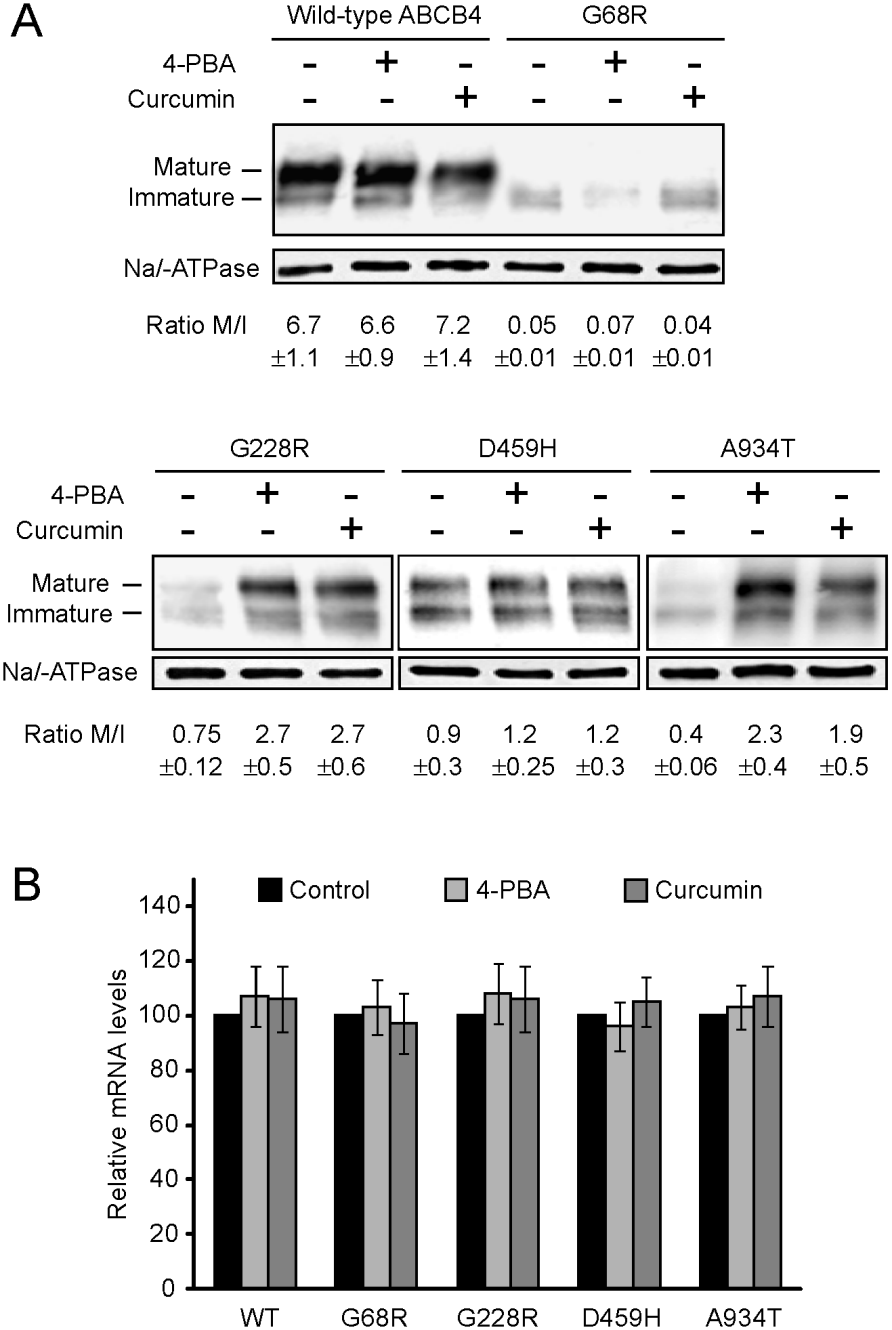
Effect of chemical chaperones on the processing of mutant ABCB4 proteins. (**A)** Western blots with anti-ABCB4 (top) and anti-Na/K-ATPase (bottom) antibodies of cell lysates (30 μg) from AD-293 transfected cells untreated or treated with 1 mM 4-PBA or 1 μM curcumin. The images are representative of four independent experiments. Numbers below the blots represent the mean±SD of the ratio between mature and immature forms of ABCB4, as determined by densitometric quantification of the signals. **(B)** Real-time quantitative PCR analysis. ABCB4 mRNA measurements were normalized to β-actin. Values represent the mean±SD of four independent experiments and are expressed relative to untreated cells (Control).

We next examined the effects of the chemical chaperones on the expression pattern of wild-type and ABCB4 mutants by western blot analysis. These experiments were conducted in non-polarized AD-293 cells, in which high and similar transfection efficiency has been achieved with the diverse *ABCB4* cDNA constructs [13,16]. As previously reported, wild-type ABCB4 was detected on western blotting as two bands of approximately 160 kDa and 140 kDa, which represent mature and immature forms of the protein, respectively [8,11,12,38]. Whole lysates from untreated AD-293 cells expressing the ABCB4 mutants exhibited markedly lower ratios of mature/immature forms with respect to those from untreated cells expressing the wild-type protein (Fig 3A). Following treatment with 4-PBA or curcumin, the abundance of the mature protein and the ratio of mature/immature forms remained unchanged for the wild-type ABCB4 and for the G68R and D459H mutants, whereas an increase in the levels of the mature form was obtained for the G228R and A934T mutants. Neither 4-PBA nor curcumin had effect on the levels of the corresponding mRNAs (Fig 3B). These results paralleled those derived from immunofluorescence studies, in which rescue of cell surface expression with any of the chemical chaperones was achieved only for the G228R and A934T mutants

**Fig 4:**
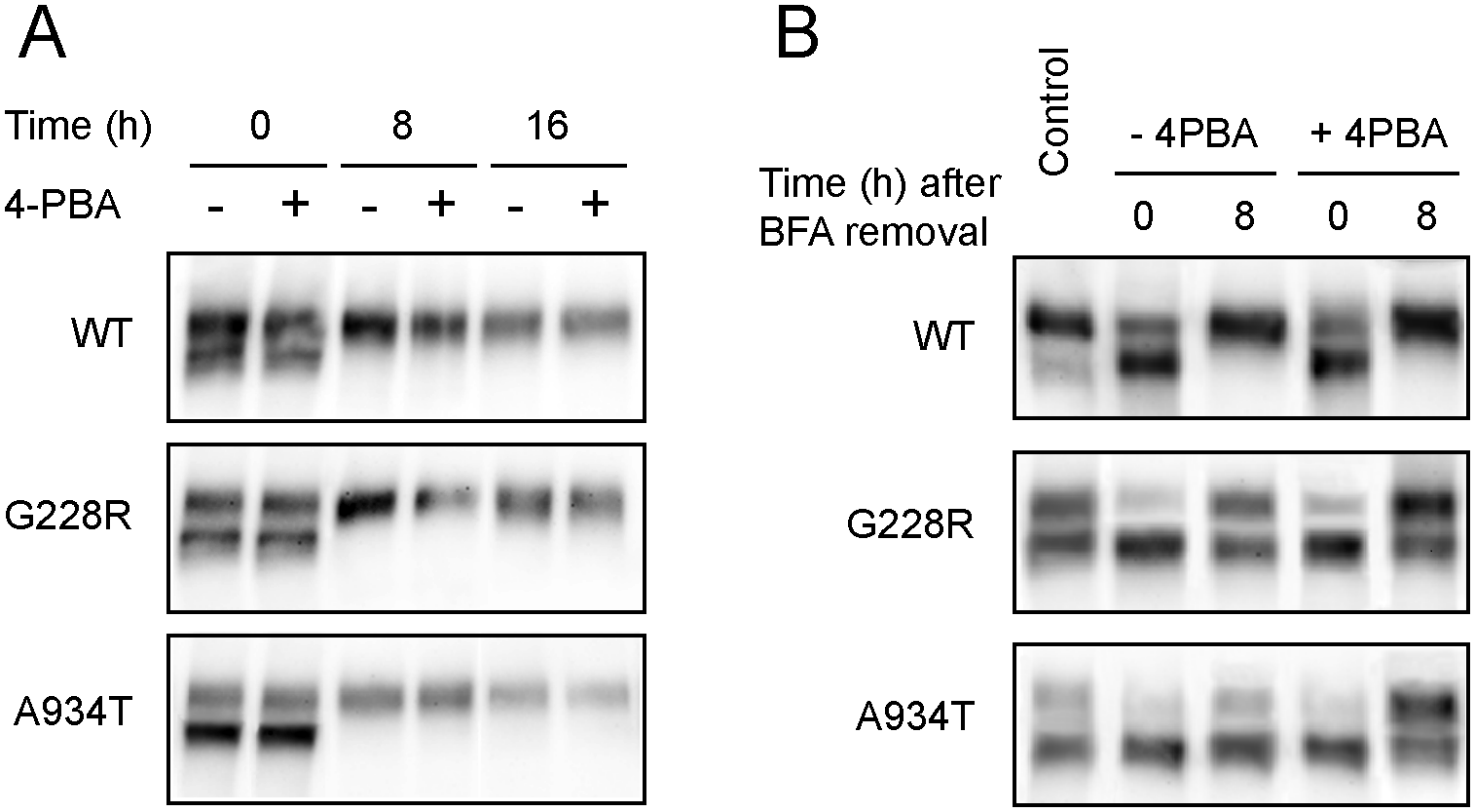
Effects of 4-PBA on the stability and maturation of the G228R and A934T mutants. **(A)** AD-293 cells expressing wild-type ABCB4 or the G228R or A934T mutants were incubated with cycloheximide (50 μg/ml) for 8 or 16 h in the presence or absence of 1mM 4-PBA. Equal amounts of proteins (30 μg) were fractionated by 6% SDS-PAGE and subjected to western blotting. **(B)** Transfected cells were untreated (control) or treated for 2 h with BFA (1 μg/ml). BFA-treated cells were subsequently rinsed and further incubated for 8 h with or without 1 mM 4-PBA. The total amount of protein loaded onto each lane was 30 μg. The images are representative of three independent experiments.

To assess whether the chemical chaperones were acting by inducing the maturation of the G228R or A934T mutants or by stabilizing their mature forms, we examined the effects of 4-PBA upon treatment with cycloheximide (CHX) or brefeldin A (BFA). As shown in Fig 4A, the inhibition of protein synthesis with CHX led to the disappearance of the immature low molecular weight form of wild-type, G228R and A934T ABCB4, and a rate of decay of the high molecular weight protein that was not influenced by 4-PBA, suggesting that this agent did not affect the stability of the mature forms of the mutants. To evaluate the effect of 4-PBA on mutants maturation, we examined the conversion of the low molecular weight protein to the high molecular weight protein after BFA treatment, which primarily inhibits protein transport from the ER to the Golgi apparatus. Cells expressing the wild-type ABCB4 or G228R or A934T mutants were pre-treated with BFA for 2 h. Then, BFA was washed out and the cells were further incubated for 8 h with or without 1 mM 4-PBA. BFA treatment resulted in a decrease of the high molecular weight protein band and an accumulation of the immature form (Fig 4B; time point 0h). Eight h after BFA removal, the wild-type ABCB4 was predominantly detected as the mature form, showing a similar pattern of expression in the presence or absence of 4-PBA. In cells expressing the G228R and A934T mutants, the fraction of the immature protein that was converted to the high molecular weight form following BFA clearance was higher in the presence of 4-PBA than in its absence (Fig 4B). This finding suggests that 4-PBA promotes the maturation of these ABCB4 mutants.

**Fig 5:**
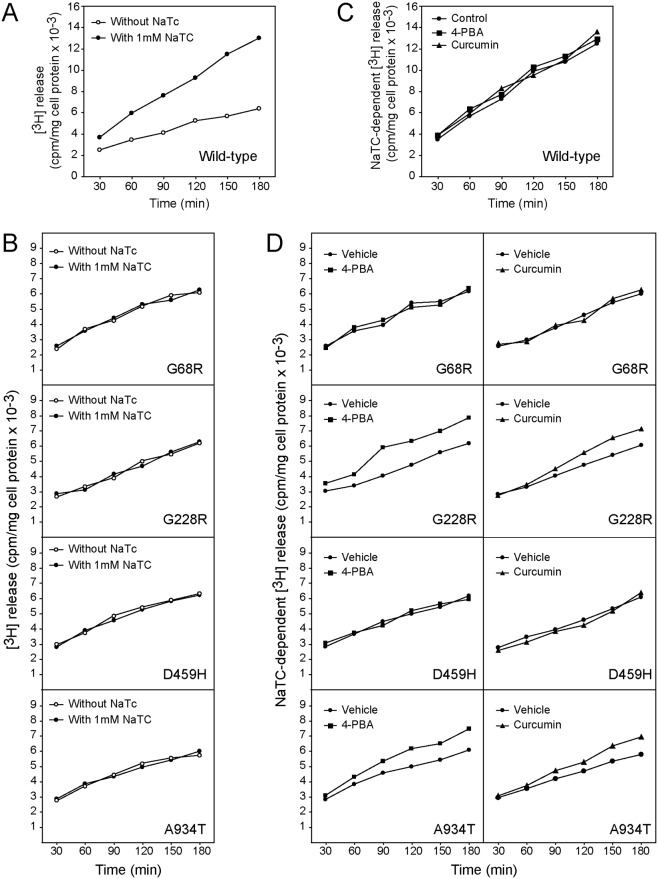
Chemical chaperones rescue phospholipid efflux activity of the ABCB4 mutants G228R and A934T. Transfected AD-293 cells were labeled with 2 μCi/mL of [3H]-choline and incubated for 3 h in EMEM with or without 1mM NaTC, as indicated. Aliquots of the culture medium were harvested each 30 min and the radioactivity released was measured by liquid scintillation counting. Values were normalized to the levels of *ABCB4* mRNA and are expressed relative to total cell protein. **(A and B)** Time course of [^3^H] release in the presence or absence of NaTC in cells expressing wild-type ABCB4 (A) or ABCB4 mutants (B). **(C)** NaTC-dependent efflux of radioactivity in cells expressing wild-type ABCB4 previously treated for 24 h with 0.1% DMSO (control), 1 mM 4-PBA, or 1 μM curcumin. **(D)** NaTC-dependent efflux of radioactivity in cells expressing ABCB4 mutants after treatment with 1 mM 4-PBA or 1 μM curcumin; H_2_O and 0.1% DMSO were used as vehicle, respectively. A representative experiment out of four is shown. For cells expressing the mutants G228R and A934T, differences in NaTC-dependent radioactivity efflux rate between vehicle- and 4-PBA- or curcumin-treated cells were statistically significant (*p* < .05).

To evaluate whether the rescued mutants were functional, we used a phospholipid efflux assay in which transfected AD-293 cells were first incubated in choline low medium, then labeled with [^3^H]-choline, and subsequently challenged with NaTC as an acceptor for PC [13]. The release of radioactivity to the cell culture supernatants was measured at various time points in the following 3 h. Fig 5A shows the result of a typical experiment performed on wild-type ABCB4-expressing cells in the presence and absence of 1mM NaTC. A bile salt-dependent increase in radioactivity efflux was achieved over time, as previously described [13,16]. The source of [^3^H]-choline released into the culture medium in response to NaTC was analyzed by examining water- and lipid-soluble extracts as detailed in Materials and Methods. The amount of radioactivity associated with choline-containing water-soluble compounds was very small (less than 5% of the total radioactivity). In the organic phase, no labeled lipids other than PC and sphingomyelin (SM) were detected. The ratio of labeled PC to labeled SM was approximately 25:1. Thus, the radioactivity in such medium consisted primarily of [^3^H]-PC.

In cells expressing the ABCB4 mutants, there were no differences in the release of [^3^H]-choline phospholipids after challenging or not with NaTC, indicating a complete absence of ABCB4 function (Fig 5B). The phospholipid efflux activity of the wild-type protein and the four ABCB4 mutants upon exposure of transfected cells to the chaperone drugs is shown in Fig 5C and 5D. The time course of radioactivity release did not change when the cells expressing wild-type ABCB4 were previously treated for 24 h with either 1 mM 4-PBA or 1 μM curcumin (Fig 5C), a result that indicates these agents have no effect on the normal floppase activity of ABCB4. In agreement with the finding that mislocalization of G68R and D459H mutants remained in the presence of the chaperone drugs, no differences in the NaTC-dependent efflux of [^3^H]-choline phospholipids were detected between untreated and treated cells expressing these mutants. In contrast, an increase in NaTC-dependent [^3^H]-choline phospholipid release was observed in cells expressing G228R and A934T mutants after treatment with either 4-PBA or curcumin (Fig 5D). Thus, restored cell surface expression of these two ABCB4 mutants resulted in a restoration of function.

## Discussion

*ABCB4* missense mutations that prevent normal trafficking of ABCB4 to the canalicular membrane of the hepatocyte result in a complete loss of ABCB4 function, which leads to progressive cholestatic liver disease. For patients carrying such mutations, liver transplantation represents the only curative option. Chaperone drugs have been tested as a therapeutic tool to correct abnormal folding or trafficking of other membrane proteins mutated in liver disease [1,39]. In the current study, we analyzed the potential therapeutic effect of such drugs on four ABCB4 mutants that display retention in the ER. Our data show that two of these four mutant proteins are prone to be rescued. Thus, the G228R and A934T mutants were efficiently targeted to the plasma membrane when folding was improved at a reduced temperature or following treatment with pharmacological doses of either 4-PBA or curcumin. The delivery of these mutants to the cell surface correlated with a switch in the ratio of mature to inmature ABCB4 forms in favor of the mature protein, an effect that can be attributed to the promotion of the maturation rate, as deduced from treatments with CHX and BFA.

It is intriguing that some amounts of the mature protein were detected by western blotting in cells expressing the D459H, G228R and A934T mutants, since no cell surface staining was observed for any of these mutants. The possibility exists that such high molecular weight protein band corresponds to a glycosylated but misfolded fraction of the mutant protein, which might be recognized and retained by the ER quality control but fail to enter the degradation pathway, as has been described for other mutant proteins [40,41].

Consistent with the recovery of cell surface expression, an increase in NaTC-dependent phospholipid efflux was observed in cells expressing G228R and A934T mutants after the addition of 4-PBA or curcumin. It can therefore be concluded that these mutants retain normal function or maintain some degree of activity, and their ability to flop PC can be recovered through restoration of membrane localization.

The effect of the chaperone drugs was mutant-specific, as G68R and D459H mutants could not be rescued even at high concentrations of either 4-PBA or curcumin. The respective mutations likely result in severely misfolded proteins or lead to an altered conformation refractory to chaperone treatment. In support of this, low-temperature incubation of cells expressing these mutants also failed to recover normal protein localization in the plasma membrane. Nevertheless, the possibility of a pharmacological rescue of these mutants cannot be completely ruled out. Gautherot et al [42] found that retention of the ABCB4 mutant I541F in the ER was not rescued by 4-PBA or the calcium-affecting drug thapsigargin, but it was by cyclosporin A. Andress et al [11] also showed that cyclosporin A was efficient in restoring apical localization of the mutant A953D. A similar effect has been recently reported for the mutants L556R and Q855L upon treatment with ciclosporins A, B, C, D and H [17]. The use of this immunosuppressant, however, does not seem to be a suitable approach to overcome such defects, given it also inhibits ABCB4 floppase activity [11].

There was a trend toward greater functional rescue of mutants G228R and A934T with 4-PBA than with curcumin at clinically feasible concentrations. This cannot be attributed to a role of 4-PBA in regulating ABCB4 activity, because no noticeable effects on NaTC-dependent phospholipid efflux were observed after the treatment of cells expressing the wild-type protein (Fig 5B). In keeping with such an observation, 4-PBA has been reported to be more effective than curcumin in rescuing selected mutants of the copper transporter ATP7B [28]. Whether this differing efficiency is related to a distinct mode of action of each drug is uncertain. In fact, the precise mechanism by which 4-PBA and curcumin facilitate the release of ER-retained mutants is as yet unknown. Several studies have suggested that 4-PBA modulates the expression of endogenous chaperones or acts as a chaperone itself [43], whereas curcumin appears to improve trafficking to the plasma membrane by altering intracellular calcium levels [44], which is thought to induce ER chaperones [45].

Both 4-PBA and curcumin are clinically approved. Curcumin is a polyphenol with pleiotropic activities. It is safe and well tolerated, but its poor bioavalability is a major limitation to its therapeutic utility [46]. 4-PBA is currently used to treat urea cycle disorders, due to its properties as an ammonia scavenger [43]. It has also been assayed for a wide range of clinical applications, including thalassemia, cystic fibrosis and cancer [47]. Its ability to treat progressive cholestatic liver disease associated with trafficking defects in canalicular membrane proteins has recently been demonstrated in selected patients with BSEP deficiency or ATP8B1 deficiency [31–35]. The current work provides experimental evidence for considering 4-PBA as an alternative treatment to liver transplantation also in patients with severe ABCB4 deficiency due to specific *ABCB4* mutations. However, as a limitation of this study, it must be taken into account that 4-PBA may act differently in distinct cells types [43], and human hepatocytes might respond in a different way. It must also be noted that our experimental approach does not allow to determine the degree of functional recovery achieved upon treatment with the chaperone drugs, although it has become evident that even a small percentage of ABCB4 activity might suffice to ameliorate disease symptoms [6]. A partial rescue of ABCB4 function might also render an adequate biliary secretion of PC to meet the requirements for a favorable response to UDCA therapy.
